# Recovery-Based Error Estimator for Natural Convection Equations Based on Defect-Correction Methods

**DOI:** 10.3390/e24020255

**Published:** 2022-02-09

**Authors:** Lulu Li, Haiyan Su, Xinlong Feng

**Affiliations:** College of Mathematics and System Sciences, Xinjiang University, Urumqi 830046, China; lulisay@stu.xju.edu.cn (L.L.); shymath@163.com (H.S.)

**Keywords:** defect-correction method, natural convection equations, adaptive methods, error bound

## Abstract

In this paper, we propose an adaptive defect-correction method for natural convection (NC) equations. A defect-correction method (DCM) is proposed for solving NC equations to overcome the convection dominance problem caused by a high Rayleigh number. To solve the large amount of computation and the discontinuity of the gradient of the numerical solution, we combine a new recovery-type posteriori estimator in view of the gradient recovery and superconvergent theory. The presented reliability and efficiency analysis shows that the true error can be effectively bounded by the recovery-based error estimator. Finally, the stability, accuracy and efficiency of the proposed method are confirmed by several numerical investigations.

## 1. Introduction

Natural convection (NC) equations for buoyancy-driven fluid often appear in practical problems. The stationary NC equation is a coupling equation for the incompressible flow and heat transfer process of viscous fluid, in which the incompressible fluid can be characterized by Boussinesq’s approximation. In atmospheric dynamics, it is an important forced dissipative nonlinear system. It contains the velocity field, pressure and temperature, which we can analyze from the thermodynamic point of view. The motion viscosity of the fluid produces a certain amount of heat, so the motion of the fluid must be accompanied by the conversion of temperature, velocity and pressure. Therefore, the study of this nonlinear system is of great significance. Christie and Mitchell [1], Boland and Layton [2,3] and others have extensively researched the numerical analysis and numerical results of NC equations (see [4,5,6,7,8,9]). In recent years, there has been continuous research on NC equations, such as in [8,10,11,12,13], which studied the natural convection of cavity-filled nanofluids. Ahmad [14] studied the effect of viscosity and thermal conductivity on natural convection in exothermic catalytic chemical reactions on curved surfaces, and in [10,15,16,17,18,19], Singh et al. considered the effect of factors such as Lorentz force on natural convection.

Combined with previous research results, the dimensionless parameter Ra (Rayleigh number) plays an important role in NC equations. It is well known that the buoyancy force is the driving force of NC equations, where the buoyancy force is the density difference caused by the temperature difference. The Rayleigh number Ra represents the ratio of the buoyancy force to the viscous force, which characterizes the relative strength of the buoyancy-driven inertial force to that of the viscous force [20,21]. When Ra≫1, the buoyancy force is much larger than the viscous force, and the convection caused by the buoyancy force is significant, which leads to the convection dominance problem [22]. Therefore, there are a number of effective numerical techniques dedicated to solving this difficulty: the variational multiscale method in [23], the defect-correction method in [24,25,26,27], etc. Among them, defect correction is one of the commonly used methods to address large Ra number problems.

The defect-correction method (DCM) is an iterative improvement technique for improving the accuracy of computational solutions without introducing mesh refinement [28,29,30,31]. In general, the basic idea can be briefly described as follows. Assume that *M* is a mapping M:X→Y, where *X* and *Y* are typical linear spaces, and assume that our goal is to find a good approximation of $x_{1}$, where $M(x_{1})=0$. Assume that we actually solve a related problem $M_{1}\left(x\right)=0$, whose solution we denote by $x_{0}$. Then, we find the residual or defect $d_{0}=M\left(x_{0}\right)$ and use it to solve the error or correct $e_{0}$, either by nonlinearly updating $M_{1}\left(x_{2}\right)-M_{1}\left(x_{0}\right)=d_{0}$ or by linearly updating $M_{1}^{\prime }\left(x_{0}\right)e_{0}=d_{0}$ to obtain the corrected solution $x_{2}$, setting $x_{2}=x_{0}+e_{0}$; obviously, this process can be repeated. If the mapping *M* is nonlinear, then the linearization of $M_{1}$ is usually chosen in the correction phase to simplify the computation. In the case of solving linear systems, the well-known iterative refinement method is an example of a defect-correction technique.

For computational practicality, the minimum-order coordinated velocity, pressure and temperature finite elements with $P_{1}-P_{0}-P_{1}$ are used to discretize NC equations. The finite element approximation of the pseudo-stress tensor sigma=Pr∇u−pI+κdiag(∇T) is the discontinuous slice constant of ${sigma}_{h}{=Pr\nabla u}_{h}-p_{h}I+\kappa diag({\nabla T}_{h}).$ We use the superconvergent slice to recover the amount of continuous space $G(\sigma_{h}).$ The approximate solution computed by the local recovered error estimator $\|\sigma_{h}-G(\sigma_{h}{\|}_{K}$ is closer to the true solution than the approximate solution of the general finite element method. The a posteriori error has been extensively studied (see [32,33,34,35,36,37)] Therefore, we use the adaptive defect-correction method to solve NC equations.

This paper is divided into four parts. First, it introduces the main properties of NC equations and the classical results. The second part proposes the defect-correction method and presents the analysis of its error estimation. The third part is combined with the restorative error estimator to solve NC equations. Finally, the validity and reliability of our method are verified by numerical experiments.

## 2. Preliminaries

Suppose that Ω=[0,1]×[0,1] is a bounded, convex open area whose boundary ∂Ω is Lipschitz continuous, so $\Gamma_{T}=\partial \Omega \setminus \Gamma_{B}$, where $\Gamma_{B}$ is a regular open subset of ∂Ω. We consider the following NC equations [7,9]:

(1) $$\left\{\begin{array}{cc} -Pr\Delta u+(u\cdot \nabla )u+\nabla p=PrRajT, & \mathrm{in}\;\;\Omega ,\\ \nabla \cdot u=0, & \mathrm{in}\;\;\Omega ,\\ -\kappa \Delta T+u\cdot \nabla T=\gamma , & \mathrm{in}\;\;\Omega ,\\ u=0,\mathrm{in}\;\;\partial \Omega ,\\ T=0,\mathrm{in}\;\;\Gamma_{T},\;\;\frac{\partial T}{\partial n}=0\;\;\mathrm{in}\;\;\Gamma_{B}, \end{array}\right.$$

where $u=(u_{1}\left(x\right),u_{2}\left(x\right))$ is fluid velocity, p=p(x) is fluid pressure, T(x) is temperature, γ is the external force function, Pr and Ra>0 are the Prandtl number and Rayleigh number, respectively, κ>0 is the thermal conductivity parameter, and $j={\left(0,1\right)}^{T}$ is the two-dimensional unit vector. Next, we introduce some Hilbert spaces:

$$X=H_{0}^{1}{\left(\Omega \right)}^{2},\;\;W=\left\{s\in H^{1}\left(\Omega \right):s=0\;\mathrm{on}\;\Gamma_{T}\right\},\;\;\;\;M=L_{0}^{2}\left(\Omega \right)=\left\{q\in L^{2}\left(\Omega \right):\int qdx=0\right\}.\;\;\;\;\;\;\;\;\;\;\;\;\;$$

We denote the inner product and norm of $L^{2}\left(\Omega \right)$ by (·,·) and $\left|\right|\cdot {\left|\right|}_{0}$. We define the inner product in space *X* and *W*. $H^{m,2}\left(\Omega \right)$ and its norm $\left|\right|\cdot {\left|\right|}_{m}$ are written as $H^{m}\left(\Omega \right)$ and $\left|\right|\cdot {\left|\right|}_{m,2}$. In addition, the dual space for $H_{0}^{1}\left(\Omega \right)$ is $H^{\prime }\left(\Omega \right)$. The dual space of *X* is $X^{\prime }$ [2].

For simplicity, we define *u* and *v* such that they belong to the same finite element space *X*. We define two continuous bilinear forms a(·,·) and d(·,·) and a trilinear form b(u;v,w):

$$\begin{array}{c} a(u,v)=(\nabla u,\nabla v),\;\;\forall u,v\in X,\\ d(v,q)=(q,\nabla \cdot v),\;\;\forall v\in X,\;\;\forall q\in M,\\ b\left(u;v,w\right)=\left(\left(u\cdot \nabla \right)v,w\right)+\frac{1}{2}\left(\left(\nabla \cdot u\right)v,w\right)\\ =\frac{1}{2}\left(\left(u\cdot \nabla \right)w,v\right)-\frac{1}{2}\left(\left(u\cdot \nabla \right)v,w\right),\;\;\forall u,v,w\in X. \end{array}$$

In addition, we need to define two bilinear forms $\bar{a}\left(\cdot ,\cdot \right)$ and a trilinear form $\bar{b}\left(\cdot ;\cdot ,\cdot \right)$ in W×W and X×W×W, respectively.

$$\begin{array}{ccc} \bar{a}\left(T,s\right)= & (\nabla T,\nabla s),\;\;\forall T,s\in W,\\ \bar{b}\left(u;T,s\right) & =\left(\left(u\cdot \nabla \right)T,s\right)+\frac{1}{2}\left(\left(\nabla \cdot u\right)T,s\right)\\ \; & =\frac{1}{2}\left(\left(u\cdot \nabla \right)T,s\right)-\frac{1}{2}\left(\left(u\cdot \nabla \right)s,T\right), & \forall u\in X,\;\;\forall T,s\in W. \end{array}$$

According to the bilinear form defined above and the trilinear form, the following two known conclusions are obtained [2,3]:

$$\begin{array}{c} b\left(u;v,w\right)=-b\left(u;w,v\right),\;\;|b\left(u;v,w\right)|\leq {N||\nabla u||}_{0}{\left||\nabla v|\right|}_{0}{\left||\nabla w|\right|}_{0},\;\;\forall u,v,w\in X,\\ \bar{b}\left(u;T,s\right)=-\bar{b}\left(u;s,T\right),\;\;|\bar{b}\left(u;T,s\right)|\leq \bar{N}{\left||\nabla u|\right|}_{0}{\left||\nabla T|\right|}_{0}{\left||\nabla s|\right|}_{0},\;\;\forall u\in X,\forall T,s\in W, \end{array}$$

where

$$N=\underset{u,v,w\in X}{\sup}\frac{\left|b(u;v,w)\right|}{{\left||\nabla u|\right|}_{0}{\left||\nabla v|\right|}_{0}{\left||\nabla w|\right|}_{0}},\;\;\bar{N}=\underset{u\in X,T,s\in W}{\sup}\frac{|\bar{b}\left(u;T,s\right)|}{{\left||\nabla u|\right|}_{0}{\left||\nabla T|\right|}_{0}{\left||\nabla s|\right|}_{0}}$$

are two fixed constants that depend only on Ω.

The variational form of the NC Equation (3) is as follows: solving (u,p,T)∈X×M×W, ∀(v,q,s)∈X×M×W:

(2) $$\left\{\begin{array}{c} Pra(u,v)+b(u;u,v)-d(v,p)+d(u,q)=PrRa(jT,v),\\ \kappa \bar{a}\left(T,s\right)+\bar{b}\left(u;T,s\right)=\left(\gamma ,s\right). \end{array}\right.$$

Then, there exists a unique result for the following solution [2,3].

**Theorem** **1.** *There exists at least a solution pair (u,p,T)∈(X,M,W) that satisfies (2) and*

$$\begin{array}{c} {\left||\nabla u|\right|}_{0}\leq Ra\kappa^{-1}{\left||\gamma |\right|}_{-1}{,\;\;||\nabla T||}_{0}\leq \kappa^{-1}{\left||\gamma |\right|}_{-1}, \end{array}$$

*where ${\left||\gamma |\right|}_{-1}=\underset{s\in W}{\sup}\frac{\left|(r,s)\right|}{\left||\nabla s|\right|}$; moreover, if Pr, Ra, κ and γ satisfy the following uniqueness condition:*

$$0<\delta =Pr^{-1}Ra\kappa^{-1}{\left||\gamma |\right|}_{-1}\left(N+Pr\bar{N}\kappa^{-1}\right),$$

*then the solution pair (u,p,T) of problem (2) is unique.*

## 3. Defect-Correction Method

### 3.1. The Finite Element Discrete Form of NC Equations

The DCM and the corresponding error estimates for steady-state NC equations are given in this section. For convenience, to represent different constants for different conditions, the constant *C* is independent of the grid parameter *h*, but it is always far from the infinity bound.

We apply an edge-to-edge triangulation of Ω into $\tau_{h}$, whose minimum angle $\theta_{min}$ is bounded away from zero, N(K) and N(e) to represent the union of all elements *K* and all edges *e* of the elements in $\tau_{h}$, respectively. In addition, $h_{e}$ and $h_{K}$ denote, as usual, the diameters of an edge *e* and element *K*, respectively. Then, velocity–pressure–temperature finite element spaces can be constructed on $\tau_{h}\left(\Omega \right)$. First, we define the finite element space $\left(X_{h},M_{h},W_{h}\right)$:

$$X_{h}=\left\{v_{h}\in C^{0}\left(\Omega \right)\cap X;v_{h}{|}_{K}\in P_{1}{\left(K\right)}^{2},\forall K\in \tau_{h}\right\},\;\;$$

$$M_{h}=\left\{q_{h}\in L^{2}{\left(\Omega \right)}^{2}\cap M;q_{h}{|}_{K}\in P_{0}{\left(K\right)}^{2},\forall K\in \tau_{h}\right\},$$

$$W_{h}=\left\{v_{h}\in C^{0}\left(\Omega \right)\cap W;v_{h}{|}_{K}\in P_{1}\left(K\right),\forall K\in \tau_{h}\right\},\;\;$$

where $P_{1}\left(K\right)$ represents piecewise linear polynomials on *K*, and $P_{0}\left(K\right)$ represents piecewise constant polynomials on *K*.

According to the above definition, the discrete form of the original equation is given: the solution of $\left(u_{h},p_{h},T_{h}\right)\in X_{h}\times M_{h}\times W_{h}$ satisfies the requirement

(3) $$\left\{\begin{array}{c} Pra\left(u_{h},v_{h}\right)+b\left(u_{h};u_{h},v_{h}\right)-d\left(v_{h},p_{h}\right)+d\left(u_{h},q_{h}\right)=PrRa\left(jT_{h},v_{h}\right),\\ \kappa \bar{a}\left(T_{h},s_{h}\right)+\bar{b}\left(u_{h};T_{h},s_{h}\right)=\left(\gamma ,s_{h}\right), \end{array}\right.$$

where $\left(v_{h},q_{h},s_{h}\right)\in X_{h}\times M_{h}\times W_{h}$.

For the finite element approximation (3), we present some results in [2,3] as follows.

**Theorem** **2.** *Let (u,p,T)∈X×M×W. Under the assumption of Theorem 1, the finite element solution $\left(u_{h},p_{h},T_{h}\right)\in X_{h}\times M_{h}\times W_{h}$ defined by scheme (3) satisfies*

$$\begin{array}{c} ||\nabla u_{h}{\left|\right|}_{0}\leq Ra\kappa^{-1}{\left||\gamma |\right|}_{-1},\;\;||\nabla T_{h}{\left|\right|}_{0}\leq \kappa^{-1}{\left||\gamma |\right|}_{-1}. \end{array}$$

For convenience, we define

(4) $$\begin{array}{c} F_{h}\left({\tilde{u}}_{h},\alpha \right)=Pra\left(u_{h},v_{h}\right)+b\left(u_{h};u_{h},v_{h}\right)-d\left(v_{h},p_{h}\right)+d\left(u_{h},q_{h}\right)+\kappa \bar{a}\left(T_{h},s_{h}\right)+\bar{b}\left(u_{h};T_{h},s_{h}\right)\\ -PrRa\left(jT_{h},v_{h}\right)-\left(\gamma ,s_{h}\right)=0, \end{array}$$

where ${\tilde{u}}_{h}=\left(u_{h},p_{h},T_{h}\right)\in X_{h}\times M_{h}\times W_{h}$, α:=(Pr,κ).

Since we choose an unstable finite element pair $P_{1}$–$P_{0}$–$P_{1}$, we consider the following penalty jump stabilization method:

$$S_{h}\left(p_{h},q_{h}\right)=\beta_{0}\sum_{e\in \Gamma_{h}\left(\Omega \right)}h_{e}\int_{e}{\left[p_{h}\right]}_{e}{\left[q_{h}\right]}_{e},\forall p_{h},q_{h}\in M_{h},$$

where $\Gamma_{h}$ is the set of inner edges of $\tau_{h}$, ${\left[q\right]}_{e}$ is the jump of *q* on edge *e*, and $\beta_{0}$ is a given stable parameter.

Therefore, formula (4) can be written as

(5) $$\begin{array}{c} F_{h}\left({\tilde{u}}_{h},\alpha \right)=Pra\left(u_{h},v_{h}\right)+b\left(u_{h};u_{h},v_{h}\right)-d\left(v_{h},p_{h}\right)+d\left(u_{h},q_{h}\right)+\kappa \bar{a}\left(T_{h},s_{h}\right)+\bar{b}\left(u_{h};T_{h},s_{h}\right)\\ -PrRa\left(jT_{h},v_{h}\right)-\left(\gamma ,s_{h}\right)+S_{h}\left(p_{h},q_{h}\right)=0. \end{array}$$

We assume that there exists an interpolation operator of the Clément type in the finite element space. Specifically, $R_{h}:X\mapsto X_{h}$ satisfies the following elementwise error estimate (see [24,27]). For φ∈X, there is $C_{i}=C_{i}\left(\theta_{min}\left(\Pi_{h}\left(\Omega \right)\right)\right)$, i=1,2,3, such that

(6) $$\begin{array}{c} ||\varphi -R_{h}{\varphi ||}_{W^{n-1,2}\left(K\right)}\leq C_{1}h_{K}^{2-n}{\left||\varphi |\right|}_{W^{1,2}\left(N\left(K\right)\right)},n=1,2,\\ ||\varphi -R_{h}{\varphi ||}_{L^{2}\left(K\right)}\leq C_{2}{\left||\varphi |\right|}_{L^{2}\left(N\left(K\right)\right)},\\ ||\varphi -R_{h}{\varphi ||}_{L^{2}\left(e\right)}\leq C_{3}h_{e}^{1/2}{\left||\varphi |\right|}_{W^{1,2}\left(N\left(e\right)\right)}. \end{array}$$

### 3.2. Application to Natural Convection Equations

We define $A_{h}$ and $D_{h}$ analogously:

$$\begin{array}{c} \langle A_{h}\left(\alpha \right){\tilde{u}}_{h},{\tilde{\varphi }}_{h}\rangle :=\sum_{K\in \tau_{h}\left(\Omega \right)}\left(\int_{K}Pr\nabla u_{h}\cdot \nabla \varphi dx+\int_{K}\kappa \nabla T_{h}\cdot \nabla sdx\right),\\ \langle D_{h}\left({\tilde{u}}_{h}\right),{\tilde{\varphi }}_{h}\rangle :=\langle F_{h}\left({\tilde{u}}_{h},\alpha \right)-A_{h}\left(\alpha \right){\tilde{u}}_{h},{\tilde{\varphi }}_{h}\rangle , \end{array}$$

where ${\tilde{u}}_{h}=\left(u_{h},p_{h},T_{h}\right)$, ${\tilde{\varphi }}_{h}:=\left(\varphi ,q,s\right)\in X_{h}\times M_{h}\times W_{h}$ and α:=(Pr,κ).

In this way, formula (5) can be written as

$$\begin{array}{c} F_{h}\left({\tilde{u}}_{h},\alpha \right)=A_{h}\left(\alpha \right){\tilde{u}}_{h}+D_{h}\left({\tilde{u}}_{h}\right)=0, \end{array}$$

where ${\tilde{u}}_{h}=\left(u_{h},p_{h},T_{h}\right)$, ${\tilde{\varphi }}_{h}:=\left(\varphi ,q,s\right)\in X_{h}\times M_{h}\times W_{h}$ and α:=(Pr,κ).

It is well known that Ra is a dimensionless result of the ratio of buoyancy to viscous force in NC equations. When buoyancy is far greater than viscous force, the convection phenomenon driven by buoyancy is more significant than the diffusion phenomenon caused by viscous force; then, convection dominates, and the regularity of $A_{h}\left(\alpha \right)$ is poor. To overcome this problem, let $\alpha_{0}\geq \alpha$, $A(\alpha_{0})$ be a more stabilized or regularized approximation of $A_{h}\left(\alpha \right)$. The DCM is given as follows:

First, solve ${\tilde{u}}_{h}^{1}$ to satisfy

(7) $$\begin{array}{c} F_{h}\left({\tilde{u}}_{h}^{1},\alpha_{0}\right)=A_{h}\left(\alpha_{0}\right){\tilde{u}}_{h}^{1}+D_{h}\left({\tilde{u}}_{h}^{1}\right)=0 \end{array}$$

Next, the above form is corrected by correction steps, which makes the obtained ${\tilde{u}}_{h}^{j+1}$ more closely approximate $\tilde{u}$ for j=1,2,…,N:

(8) $$\begin{array}{c} A_{h}\left(\alpha_{0}\right){\tilde{u}}_{h}^{j+1}+D_{h}\left({\tilde{u}}_{h}^{j+1}\right)=\left(A_{h}\left(\alpha_{0}\right)-A_{h}\left(\alpha \right)\right){\tilde{u}}_{h}^{j}. \end{array}$$

With $A_{h}$, α, $\alpha_{0}\left(K\right):=max\left(\alpha ,h_{K}\right)$ and $D_{h}\left(\cdot \right)$, the defect-correction discretization is applied to the incompressible NC equations (see [6]), where the trilinear term b(·;·,·) and $\bar{b}\left(\cdot ;\cdot ,\cdot \right)$ are discretized by the Oseen scheme. In the calculation process, in order to produce better results, $D_{h}\left(\cdot \right)$ is usually disturbed by the local average or flux limiters or by merging the appropriate subgrid model.

The defect-correction method for problem (3) can be described as follows:

**Algorithm** **1.** *Step 1. Find a stabilized finite element iterative solution*$\left(u_{h}^{m},p_{h}^{m},T_{h}^{m}\right)$*such that*

(9) $$\left\{\begin{array}{c} \left(Pr+ch\right)a\left(u_{h}^{m},v_{h}\right)+b\left(u_{h}^{m-1};u_{h}^{m},v_{h}\right)-d\left(v_{h},p_{h}^{m}\right)+d\left(u_{h}^{m},q_{h}\right)+S_{h}\left(p_{h}^{m},q_{h}\right)=PrRa\left(jT_{h}^{m},v_{h}\right),\\ \left(\kappa +ch\right)\bar{a}\left(T_{h}^{m},s_{h}\right)+\bar{b}\left(u_{h}^{m-1};T_{h}^{m},s_{h}\right)=\left(\gamma ,s_{h}\right) \end{array}\right.$$

*for all*$\left(v_{h},q_{h},s_{h}\right)\in \left(X_{h},M_{h},W_{h}\right)$, m=1,2,….

*Step 2. For j = 0,1,2,. . ., find a corrected solution*$\left(u_{j+1}^{h},p_{j+1}^{h},T_{j+1}^{h}\right)$*such that*

(10) $$\left\{\begin{array}{c} \left(Pr+ch\right)a\left(u_{j+1}^{h},v_{h}\right)+b\left(u_{j}^{h};u_{j+1}^{h},v_{h}\right)-d\left(v_{h},p_{j+1}^{h}\right)+d\left(u_{j+1}^{h},q_{h}\right)+S_{h}\left(p_{j+1}^{h},q_{h}\right)\\ =PrRa\left(jT_{j+1}^{h},v_{h}\right)+cha\left(u_{j}^{h},v_{h}\right),\\ \left(\kappa +ch\right)\bar{a}\left(T_{j+1}^{h},s_{h}\right)+\bar{b}\left(u_{j}^{h};T_{j+1}^{h},s_{h}\right)=\left(\gamma ,s_{h}\right)+\bar{a}\left(T_{j}^{h},s_{h}\right), \end{array}\right.$$

*with*$\left(u_{0}^{h},p_{0}^{h},T_{0}^{h}\right)=\left(u_{h}^{m},p_{h}^{m},T_{h}^{m}\right)$.

### 3.3. Error Analysis

Similarly, the term $\left|\right|R_{h}{\left|\right|}_{L(X,Y^{*})}\left|\right|\left(A\left(\alpha \right)-A_{h}\left(\alpha \right)\right)u^{j+1}+\left(D-D_{h}\right)\left(u^{j+1}\right){\left|\right|}_{Y_{h}^{*}}$ is a consistency error, which is a higher-order term, so we only need to estimate $\left|\right|{\left(I_{Y}-R_{h}\right)}^{*}\left[A\left(\alpha \right)u^{j+1}+D\left(u^{j+1}\right)\right]{\left|\right|}_{Y^{*}}$ and $\left|\right|R_{h}{\left|\right|}_{L(X,Y^{*})}\left|\right|\left(A_{h}\left(\alpha_{0}\right)-A_{h}\left(\alpha \right)\right)\left(u^{j+1}-u^{j}\right){\left|\right|}_{Y_{h}^{*}}$.

**Theorem** **3.** *Suppose that (u,p,T) and $\left(u^{j+1},p^{j+1},T^{j+1}\right)$ are the solutions of (2) and (5), respectively:*

(11) $$\begin{array}{c} ||u-u^{j+1}{\left|\right|}_{1}+||p-p^{j+1}{\left|\right|}_{0}+||T-T^{j+1}{\left|\right|}_{1}\\ \leq C||RF{\left(\tilde{u},\alpha \right)}^{-1}{\left|\right|}_{L(Y^{*},X)}\left(\sum_{K\in \tau_{h}\left(\Omega \right)}h_{K}^{2}\left(|\right|r_{1}^{j+1}{\left|\right|}_{0,K}^{2}+||\nabla \cdot u^{j+1}{\left|\right|}_{0,K}^{2}+\left|\right|r_{2}^{j+1}{\left|\right|}_{0,K}^{2})\right.\\ +\sum_{K\in \tau_{h}\left(\Omega \right)}h_{e}\left|\right|{\left[Pr\nabla u^{j+1}\cdot n_{e}-p^{j+1}n_{e}+\kappa diag\left(\nabla T^{j+1}\right)\cdot n_{e}\right]}_{e}{\left|\right|}_{0,e}\\ {\left.+\sum_{K\in \tau_{h}\left(\Omega \right)}\left|\right|\left(\alpha_{0}\left(K\right)-\alpha \right)\nabla \cdot \left(u^{j+1}-u^{j}\right){\left|\right|}_{0,K}^{2}\right)}^{1/2}, \end{array}$$

*where $RF\left(\tilde{u},\alpha \right)=A\left(\alpha \right)+RD\left(\tilde{u}\right)$, RD(·) is Lipschitz continuous in some region around $\tilde{u}$, and diag(u) is a diagonal matrix with diagonal elements from the vector u.*

**Proof** **of** **Theorem** **1.** First, we consider $\left|\right|{\left(I_{Y}-R_{h}\right)}^{*}\left[A\left(\alpha \right)u^{j+1}+D\left(u^{j+1}\right)\right]{\left|\right|}_{Y^{*}}$. We integrate by parts over each element $K\in \tau_{h}\left(\Omega \right)$ and denote the collection of edges of $\tau_{h}\left(\Omega \right)$ in the interior of Ω by $E^{h}$. Using the Cauchy–Schwarz inequality on each element *K* and edge *e*:

(12) $$\begin{array}{c} \langle \left(I_{Y}-{\tilde{R}}_{h}\right)*\left[A\left(\alpha \right){\tilde{u}}^{j+1}+D\left({\tilde{u}}^{j+1}\right)\right],\tilde{\varphi }\rangle =\langle A\left(\alpha \right){\tilde{u}}^{j+1}+D\left({\tilde{u}}^{j+1}\right),\tilde{\varphi }-{\tilde{R}}_{h}\tilde{\varphi }\rangle \\ =\sum_{K\in \tau_{h}\left(\Omega \right)}\int_{K}-r_{1}^{j+1}\cdot \nabla \left(\varphi -R_{h}^{X}\varphi \right)+\int_{K}\left(q-R_{h}^{M}q\right)\nabla \cdot u^{j+1}+\int_{K}-r_{2}^{j+1}\cdot \nabla \left(s-R_{h}^{W}s\right)dx\\ +\sum_{K\in \tau_{h}\left(\Omega \right)}\int_{e}{\left[Pr\nabla u^{j+1}\cdot n_{e}\right]}_{e}\cdot \left(\varphi -R_{h}^{X}\varphi \right)-{\left[p^{j+1}\right]}_{e}\left(\varphi -R_{h}^{X}\varphi \right)\cdot n_{e}\\ +{\left[\kappa diag\left(\nabla T^{j+1}\right)\cdot n_{e}\right]}_{e}\cdot \left(s-R_{h}^{W}s\right)d\sigma \\ \leq C{ [\sum_{K\in \tau_{h}\left(\Omega \right)}h_{K}^{2}\left(|\right|r_{1}^{j+1}{\left|\right|}_{0,T}^{2}+||\nabla \cdot u^{j+1}{\left|\right|}_{0,K}^{2}+\left|\right|r_{2}^{j+1}{\left|\right|}_{0,K}^{2})]}^{1/2}\\ +C{ [\sum_{K\in \tau_{h}\left(\Omega \right)}h_{e}\left|\right|{\left[Pr\nabla u^{j+1}\cdot n_{e}-p^{j+1}n_{e}+\kappa diag\left(\nabla T^{j+1}\right)\cdot n_{e}\right]}_{e}{\left|\right|}_{0,e}]}^{1/2}. \end{array}$$

We assume $\tilde{\varphi }=\left(\varphi ,q,s\right)\in Y:=X\times M\times W$ and $\left|\right|\tilde{\varphi }{\left|\right|}_{Y}=1$, where $r_{1}^{j+1}=PrRaT^{j+1}-\left(-Pr▵u^{j+1}+u^{j+1}\cdot \nabla u^{j+1}+\nabla p^{j+1}\right)$, $r_{2}^{j+1}=\gamma -\left(-\kappa ▵T^{j+1}+u^{j+1}\cdot \nabla T^{j+1}\right)$.

According to the usual Galerkin formulation, $\left|\right|R_{h}{\left|\right|}_{L(X,Y^{*})}\left|\right|\left(A\left(\alpha \right)-A_{h}\left(\alpha \right)\right)u^{j+1}+\left(D-D_{h}\right)\left(u^{j+1}\right){\left|\right|}_{Y_{h}^{*}}$ is identically zero. For this last term involving $A_{h}\left(\alpha_{0}\right)-A_{h}\left(\alpha \right)$, let $\tilde{\varphi }\in X\times M\times W$ satisfy $\left|\right|\tilde{\varphi }{\left|\right|}_{Y}=1$. Then,

(13) $$\begin{array}{cc} \langle \left(A_{h}\left(\alpha_{0}\right)-A_{h}\left(\alpha \right)\right)\left({\tilde{u}}^{j+1}-{\tilde{u}}^{j}\right),\tilde{\varphi }\rangle \; & =\int_{\Omega }\left(\alpha_{0}-\alpha \right)\nabla \left(u^{j+1}-u^{j}\right)\cdot \nabla \varphi dx\\ \; & \leq { [\sum_{K\in \tau_{h}\left(\Omega \right)}\left|\right|\left(\alpha_{0}\left(K\right)-\alpha \right)\nabla \cdot \left(u^{j+1}-u^{j}\right){\left|\right|}_{0,K}^{2}]}^{1/2}. \end{array}$$

This completes the proof for Theorem 1. □

Note that this amounts to replacing $||RF{\left(\tilde{u},\alpha \right)}^{-1}{\left|\right|}_{L(Y^{*},X)}$ by $||RF{\left({\tilde{u}}^{j+1},\alpha \right)}^{-1}{\left|\right|}_{L(Y^{H*},X^{H})}$, where H≫h. The residual term is $r^{j+1}$, and $C=C(C_{1},C_{2},\theta_{min}\left(\tau_{h}\left(\Omega \right)\right))$ is a computable constant. In addition, let $S_{h}$ be the set of all interior sides in Ω, and for any piecewise constant *q*, let ${\left[q\right]}_{e}=q{{|}_{K_{e}^{+}}-q|}_{K_{e}^{-}}$ denote their jumps on the side $e\in S_{h}$, where $K_{e}^{+}$ and $K_{e}^{-}$ are two triangulations sharing the common side *e*. Let $h_{e}$ be the length of edge $e\in S_{h}$, and define the edge norm as

$${\left||u|\right|}_{S_{h}}={\left(\sum_{e\in S_{h}}h_{e}{\left||u|\right|}_{e}^{2}\right)}^{\frac{1}{2}}.$$

## 4. Recovery Error Estimator for NC Equations

### 4.1. Recovery Error Estimator

In this section, we construct a recovery error estimator and analyze its properties. Assuming that $\left(u_{h},p_{h},T_{h}\right)$ is the numerical result, we consider the pseudo-stress tensor σ:=Pr∇u−pI+κdiag(∇T) and its finite element approximation $\sigma_{h}^{j+1}:=Pr\nabla u_{h}^{j+1}-p_{h}^{j+1}I+\kappa diag\left(\nabla T_{h}^{j+1}\right)$.

*I* is 2×2-identity matrix. The main idea of the Zienkienwicz–Zhu estimator is to restore the discontinuous finite element gradient to a continuous recovery term (see [37]).

In $\tau_{h}$, *N*, $N_{h}$, $\phi_{i}$ and $\omega_{v}$ are respectively defined as the set of all vertices in $\tau_{h}$, the set of all vertices within Ω in $\tau_{h}$ and the base function of *i* (∀i∈N) that shares a collection of all units of a vertex ($\omega_{i}:=\mathrm{supp}\;\phi_{i}$).

Combined with the superconvergent piece recovery technique in the literature [37], the piecewise constant tensor $\sigma_{h}^{j+1}$ is restored to continuous, and a recovery pseudo-stress tensor is obtained.

For any vertex i∈N and its patch $\omega_{i}$, the following is defined:

$$G\left(\sigma_{h}^{j+1}\right)\left(i\right)=\sum_{K\in \omega_{v}}\frac{\left|K\right|\sigma_{h}^{j+1}}{|\omega_{i}|},$$

where |K| is the area of triangulation *K*. Details can be found in [37].

A recovery error estimator is constructed. The local estimator and global estimator are defined as

$$\eta_{K}:=\left|\right|\sigma_{h}^{j+1}-G\left(\sigma_{h}^{j+1}\right){\left|\right|}_{K},\;\;\eta :={\left(\sum_{K\in \tau_{h}}\eta_{K}^{2}\right)}^{\frac{1}{2}}.$$

The recovery error estimator $\eta_{K}$ has the following important properties. The proof is similar to that in the literature ([37]) and is not presented in detail here.

Before moving to the next subsection, we illustrate the connection between $\left|\right|{\left[\sigma_{h}^{j+1}\right]}_{e}{\left|\right|}_{e}$ and $\left|\right|\left[\sigma_{h}^{j+1}\cdot n_{e}\right]{\left|\right|}_{e}$, where $n_{e}$ is the unit outward normal vector on edge *e*, which plays an important role in the a posterior error of this recovery-based error estimator. In two dimensions, $\iota_{e}$ denotes the unit tangential vector along *e*.

$$\left[\sigma \cdot n_{e}\right]:=\sigma {{|}_{K_{e}^{+}}\cdot n_{e}+\sigma |}_{K_{e}^{-}}\cdot n_{e},$$

$$\left[\sigma \cdot \iota_{e}\right]:=\sigma {{|}_{K_{e}^{+}}\cdot \iota_{e}+\sigma |}_{K_{e}^{-}}\cdot \iota_{e}$$

denote the jumps of the normal and tangential component of $\sigma_{h}^{j+1}$ on side *e*, respectively. Since $[\nabla u_{h}^{j+1}\cdot n_{e}]=0$, we have

$${\left[\sigma_{h}^{j+1}\right]}_{e}=\left(\left[\sigma_{h}^{j+1}\cdot n_{e}\right],\left[\nabla u_{h}^{j+1}\cdot \iota_{e}\right]+-p_{h}^{j+1}I\iota_{e}]+\left[\nabla u_{h}^{j+1}\cdot \iota_{e}\right]\right)$$

$$=(\left[\sigma_{h}^{j+1}\cdot n_{e}\right],\left[-p_{h}^{j+1}I\iota_{e}\right]+\left[\nabla T_{h}^{j+1}\cdot \iota_{e}\right]).$$

Therefore,

$$\left|\right|{\left[\sigma_{h}^{j+1}\right]}_{e}{\left|\right|}_{e}^{2}=\left|\right|\left[\sigma_{h}^{j+1}\cdot n_{e}\right]{\left|\right|}_{e}^{2}+\left|\right|{\left[p_{h}^{j+1}\right]}_{e}+[\nabla T_{h}^{j+1}]\cdot \iota_{e}{\left|\right|}_{e}^{2}.$$

From the above relation, we have

$$\left|\right|\left[\sigma_{h}^{j+1}\cdot n_{e}\right]{\left|\right|}_{S_{h}}\leq \left|\right|{\left[\sigma_{h}^{j+1}\right]}_{e}{\left|\right|}_{S_{h}}\leq c\eta_{R},$$

$$\left|\right|{\left[p_{h}^{j+1}\right]}_{e}{\left|\right|}_{S_{h}}\leq \left|\right|{\left[\sigma_{h}^{j+1}\right]}_{e}{\left|\right|}_{S_{h}}\leq c\eta_{R},$$

$$\left|\right|\left[\nabla T_{h}^{j+1}\cdot \iota_{e}\right]{\left|\right|}_{S_{h}}\leq \left|\right|{\left[\sigma_{h}^{j+1}\right]}_{e}{\left|\right|}_{S_{h}}\leq c\eta_{R}.$$

**Lemma** **1.** *Based on the definitions of $\sigma_{h}^{j+1}$ and $G(\sigma_{h}^{j+1})$, there are two grid-independent constants $C_{1}$ and $C_{2}$.*

$$\begin{array}{c} C_{1}\left(|\right|\left[\sigma_{h}^{j+1}\cdot n_{e}\right]{\left|\right|}_{S_{h}}+\left|\right|{\left[p_{h}^{j+1}\right]}_{e}{\left|\right|}_{S_{h}}+\left|\right|{\left[\nabla T_{h}^{j+1}\right]}_{e}{\left|\right|}_{S_{h}})\\ \leq \left|\right|\sigma_{h}^{j+1}-G\left(\sigma_{h}^{j+1}\right)\left|\right|\leq C_{2}\left(|\right|\left[\sigma_{h}^{j+1}\cdot n_{e}\right]{\left|\right|}_{S_{h}}+\left|\right|{\left[p_{h}^{j+1}\right]}_{e}{\left|\right|}_{S_{h}}+\left|\right|{\left[\nabla T_{h}^{j+1}\right]}_{e}{\left|\right|}_{S_{h}}). \end{array}$$

**Lemma** **2.** *According to the definitions of $\sigma_{h}^{j+1}$ and $G(\sigma_{h}^{j+1})$, there is a constant C independent of the grid, and the following $\left|\right|\left[\nabla u_{h}^{j+1}\cdot n_{e}\right]{\left|\right|}_{S_{h}}$ and $\left|\right|{\left[\nabla T_{h}^{j+1}\right]}_{e}{\left|\right|}_{S_{h}}$ error estimation can be obtained.*

$$\begin{array}{c} C(||\left[\nabla u_{h}^{j+1}\cdot n_{e}\right]{\left|\right|}_{S_{h}}+\left|\right|{\left[p_{h}^{j+1}\right]}_{e}{\left|\right|}_{S_{h}}+\left|\right|{\left[\nabla T_{h}^{j+1}\right]}_{e}{\left|\right|}_{S_{h}})\leq ||\left[\nabla \sigma_{h}^{j+1}\cdot n_{e}\right]{\left|\right|}_{S_{h}}+\left|\right|{p_{h}^{j+1}]}_{e}{\left|\right|}_{S_{h}}. \end{array}$$

**Proof** **of** **Lemma** **2.** According to the Brezis–Gallonet inequality in [37] and the method in [37], the conclusion can be easily obtained. □

### 4.2. The Reliability Analysis

A basic framework of posteriori error estimation for nonlinear stability problems is proposed, and the basic equivalence of the error to residual error is established. We introduce the main results and apply them to estimate the restorative error estimator.

**Theorem** **4.** *Suppose that (u,p,T) and $\left(u_{h}^{j+1},p_{h}^{j+1},T_{h}^{j+1}\right)$ are the solutions of (2) and (5), respectively,*

(14) $$||\nabla \left(u-u_{h}^{j+1}\right){\left|\right|}_{0}+||p-p_{h}^{j+1}{\left|\right|}_{0}+||\nabla \left(T-T_{h}^{j+1}\right){\left|\right|}_{0}\leq C\eta .$$

**Proof** **of** **Theorem** **2.** According to (12), with the Cauchy–Schwarz inequality, we have

(15) $$\begin{array}{c} \langle \left(I_{Y}-{\tilde{R}}_{h}\right)*\left[A\left(\alpha \right){\tilde{u}}^{j+1}+D\left({\tilde{u}}^{j+1}\right)\right],\tilde{\varphi }\rangle \\ \leq C\left(\sum_{K\in \tau_{h}\left(\Omega \right)}h_{K}^{2}\left|\right|r_{1}^{j+1}{\left|\right|}_{0,K}^{2}+||\nabla \cdot u_{h}^{j+1}{\left|\right|}_{0,K}^{2}+h_{K}^{2}\left|\right|r_{2}^{j+1}{\left|\right|}_{0,K}^{2}\right.\\ {\left.+\sum_{K\in \tau_{h}\left(\Omega \right)}h_{e}\left|\right|{\left[Pr\nabla u_{h}^{j+1}\cdot n_{e}-p_{h}^{j+1}n_{e}+\kappa diag\left(\nabla T_{h}^{j+1}\right)\cdot n_{e}\right]}_{e}\right)}^{1/2}\\ \leq C\left(\sum_{K\in \tau_{h}\left(\Omega \right)}h_{K}^{2}\left|\right|r_{1}^{j+1}{\left|\right|}_{0,K}^{2}+||\nabla \cdot u_{h}^{j+1}{\left|\right|}_{0,K}^{2}+h_{K}^{2}\left|\right|r_{2}^{j+1}{\left|\right|}_{0,K}^{2}\right.\\ {\left.+\sum_{K\in \tau_{h}\left(\Omega \right)}h_{e}\left|\right|\sigma_{h}^{j+1}\cdot n_{e}{\left|\right|}_{0,K}^{2}\right)}^{1/2}\\ \leq C\eta . \end{array}$$

According to the definition of $B_{h}$, the approximate solution $\left(u_{h}^{j+1},p_{h}^{j+1},T_{h}^{j+1}\right)$ satisfies

$${\langle F_{h}\left({\tilde{u}}_{h}^{j+1}\right),\tilde{\varphi }\rangle }_{X_{h}\times M_{h}\times W_{h}}=0.$$

At the same time ${\langle F_{h}\left({\tilde{u}}_{h}^{j+1}\right),\tilde{\varphi }\rangle }_{X\times M\times W}=0$.

With the above lemma, we obtain

$$||\nabla \left(u-u_{h}^{j+1}\right){\left|\right|}_{0}+||p-p_{h}^{j+1}{\left|\right|}_{0}+||\nabla \left(T-T_{h}^{j+1}\right){\left|\right|}_{0}\leq C\eta .$$

Theorem 4 is proved. □

### 4.3. Effectiveness Analysis

From Lemma 1, the following can be obtained:

$$\eta \leq C(||\left[\sigma_{h}^{j+1}\cdot n_{e}\right]|{|}_{S_{h}}+||{\left[p_{h}^{j+1}\right]}_{e}|{|}_{S_{h}}+||{\left[\nabla T_{h}^{j+1}\right]}_{e}|{|}_{S_{h}}).$$

In order to prove the validity of the recovery error estimator η, we first need to estimate $\left|\right|\left[\sigma_{h}^{j+1}\cdot n_{e}\right]{\left|\right|}_{S_{h}},\;||{\left[p_{h}^{j+1}\right]}_{e}{\left|\right|}_{S_{h}}$ and $\left|\right|{\left[\nabla T_{h}^{j+1}\right]}_{e}{\left|\right|}_{S_{h}}.$

**Lemma** **3.** *Suppose that (u,p,T) and $\left(u_{h}^{j+1},p_{h}^{j+1},T_{h}^{j+1}\right)$ are the solutions of (2) and (5), respectively. There is a normal number C that is not related to the grid:*

$$\left|\right|\left[\sigma_{h}^{j+1}\cdot n_{e}\right]{\left|\right|}_{S_{h}}\leq C||\nabla \left(u-u_{h}^{j+1}\right){\left|\right|}_{0}+||p-p_{h}^{j+1}{\left|\right|}_{0}+||\nabla \left(T-T_{h}^{j+1}\right){\left|\right|}_{0}.$$

**Proof** **of** **Lemma** **3.** From the definition of $\sigma_{h}$, the following can be obtained:

(16) $$\begin{array}{c} \left|\right|\left[\sigma_{h}^{j+1}\cdot n_{e}\right]{\left|\right|}_{S_{h}}=\left|\right|\left[\left(\nabla u_{h}^{j+1}-p_{h}^{j+1}I+\nabla T_{h}^{j+1}\right)\cdot n_{e}\right]{\left|\right|}_{S_{h}}\\ \leq C||\left[\left(\nabla u_{h}^{j+1}-p_{h}^{j+1}I\right)\cdot n_{e}\right]{\left|\right|}_{S_{h}}+C||\left[\nabla T_{h}^{j+1}\cdot n_{e}\right]{\left|\right|}_{S_{h}}\\ \leq C||\nabla \left(u-u_{h}^{j+1}\right){\left|\right|}_{0}+||p-p_{h}^{j+1}{\left|\right|}_{0}+||\nabla \left(T-T_{h}^{j+1}\right){\left|\right|}_{0}. \end{array}$$

Specific details can be found in [37]. □

Next, we define the operator Π: $L^{2}\left(\Omega \right)\to R_{1,h}\left(\Omega \right)$.

$$\begin{array}{c} \left|\right|{\left[\nabla T_{h}^{j+1}\right]}_{e}{\left|\right|}_{S_{h}}\leq \left|\right|{\left[\nabla T_{h}^{j+1}-\Pi \nabla T\right]}_{e}{\left|\right|}_{S_{h}}\leq C(||\nabla T-\nabla T_{h}^{j+1}\left|\right|+||\nabla T-\Pi \nabla T||)\\ \;\;\;\;\;\;\;\;\;\;\;\;\;\;\;\;\;\;\leq C||\nabla \left(u-u_{h}^{j+1}\right){\left|\right|}_{0}+||p-p_{n}^{h}{\left|\right|}_{0}+||\nabla \left(T-T_{h}^{j+1}\right){\left|\right|}_{0}.\\ \left|\right|{\left[p_{h}^{j+1}\right]}_{e}{\left|\right|}_{S_{h}}\leq \left|\right|{\left[p_{h}^{j+1}-\Pi p\right]}_{e}{\left|\right|}_{S_{h}}\leq C(||p-p_{h}^{j+1}\left|\right|+||p-\Pi p||)\\ \;\;\;\;\;\;\;\;\;\;\;\;\;\;\;\;\leq C||\nabla \left(u-u_{h}^{j+1}\right){\left|\right|}_{0}+||p-p_{h}^{j+1}{\left|\right|}_{0}+||\nabla \left(T-T_{h}^{j+1}\right){\left|\right|}_{0}. \end{array}$$

**Theorem** **5.** *Suppose that (u,p,T) and $\left(u_{h}^{j+1},p_{h}^{j+1},T_{h}^{j+1}\right)$ are the solutions of (2) and (5), respectively, and C is a grid-independent constant.*

$$\eta \leq C||\nabla \left(u-u_{h}^{j+1}\right){\left|\right|}_{0}+||p-p_{h}^{j+1}{\left|\right|}_{0}+||\nabla \left(T-T_{h}^{j+1}\right){\left|\right|}_{0}.$$

**Proof** **of** **Theorem** **3.** Combined with the above estimates, Lemma 1 and Lemma 3 are applied.

$$\begin{array}{c} \eta \leq C(||\left[\sigma_{h}^{j+1}\cdot n_{e}\right]|{|}_{S_{h}}+||{\left[p_{h}^{j+1}\right]}_{e}|{|}_{S_{h}}+||{\left[\nabla T_{h}^{j+1}\right]}_{e}|{|}_{S_{h}})\;\;\;\;\;\;\;\;\;\;\;\;\;\;\;\\ \leq C||\nabla \left(u-u_{h}^{j+1}\right){\left|\right|}_{0}+||p-p_{h}^{j+1}{\left|\right|}_{0}+||\nabla \left(T-T_{h}^{j+1}\right){\left|\right|}_{0}.\;\;\;\;\;\;\;\;\;\;\; \end{array}$$

The proof of validity is complete. □

## 5. Numerical Experiment

In this section, four numerical examples are given to verify the effectiveness of the proposed method combined with the adaptive method.

For the sake of convenience, a few definitions are given below:

- DOF:= the number of degrees of freedom of triangulation $\tau_{h}^{j}$;
- $e_{r}:=\frac{(||u-u_{h}{\left|\right|}_{1}^{2}+||p-p_{h}{\left|\right|}_{0}^{2}+||T-T_{h}{\left|\right|}_{1}^{2}{)}^{\frac{1}{2}}}{{\left(||u|\right|}_{1}^{2}+{\left||p|\right|}_{0}^{2}+{\left||T|\right|}_{1}^{2}{)}^{\frac{1}{2}}}$ denotes the relative value of $L^{2}$ norm;
- $\eta_{r}:=\frac{\eta }{{\left(||u|\right|}_{1}^{2}+{\left||p|\right|}_{0}^{2}+{\left||T|\right|}_{1}^{2}{)}^{\frac{1}{2}}}$ denotes the relative value of global recovery-based estimator;
- $e_{r}rate:=\frac{2\log(e_{r}^{j+1}/e_{r}^{j})}{\log(DOF^{j}/DOF^{j+1})}$ denotes the convergence rate of the error;
- $\eta_{r}rate:=\frac{2\log(\eta_{r}^{j+1}/\eta_{r}^{j})}{\log(DOF^{j}/DOF^{j+1})}$ denotes the convergence rate of the error;
- $I_{eff}:=\frac{\eta_{r}}{e_{r}}$ denotes effectivity index for the global recovery-based estimator $\eta_{r}$,

where the validity index $I_{eff}$ denotes the ratio of the estimator error to the true error $\eta_{r}/e_{r}$, and if $I_{eff}$ tends to 1, it means that our estimator error is asymptotically equivalent to the true error, thus verifying our validity and reliability.

### 5.1. Smooth True Solution

The purpose of the first example is to solve a smooth true solution problem in the $\Omega ={\left[0,1\right]}^{2}$ region and verify our method, which is effective for the smooth true solution. We define the true solution $u=(u_{1},u_{2})$, pressure *p* and temperature *T* as follows:

$$\begin{array}{c} u_{1}\left(x_{1},x_{2}\right)=10x_{1}^{2}{\left(x_{1}-1\right)}^{2}x_{2}\left(x_{2}-1\right)\left(2x_{2}-1\right),\\ u_{2}\left(x_{1},x_{2}\right)=-10x_{1}\left(x_{1}-1\right)\left(2x_{1}-1\right)x_{2}^{2}{\left(x_{2}-1\right)}^{2},\\ p\left(x_{1},x_{2}\right)=10\left(2x_{1}-1\right)\left(2x_{2}-1\right),\\ T\left(x_{1},x_{2}\right)=u_{1}\left(x_{1},x_{2}\right)+u_{2}\left(x_{1},x_{2}\right). \end{array}$$

**Figure 1:**
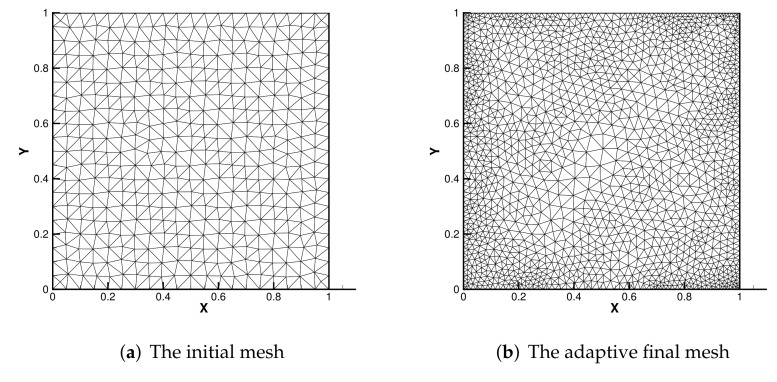
The initial mesh and adaptive final mesh.

Figure 1 shows the initial mesh of the adaptive method and the adaptive grid. From Table 1 and Table 2, if only the defect-correction method is used to solve the NC equations, more degrees of freedom are needed to achieve better accuracy, while the adaptive method can yield better accuracy with less vertex information. For example, the accuracy of 5000 degrees of freedom in Table 2 is similar to that of 2773 degrees of freedom in Table 1. This shows that the adaptive method is more economical. Table 3 presents the comparison of the results of our method (DCM) and the adaptive algorithm without the defect-correction method (NDCM). We selected the values of the error estimator $\eta_{r}$ for similar degrees of freedom with different Ra. From the table, we can see that the error results without the defect-correction method when $Ra=10^{4}$ are very large, and when Ra is greater than $10^{4}$, the results are not counted. Our method, on the other hand, is still relatively stable at $Ra=10^{5}$. It can be seen that the posteriori error estimator based on the defect-correction method is applicable. $I_{eff}$ tends to 1, which indicates that our estimators and real errors are effective and progressive, indicating that our method is effective.

### 5.2. L-Shape Domain Problem

The second example is a flow problem in the L-shape domain $\Omega ={\left(-1,1\right)}^{2}-{\left[0,1\right]}^{2}$, with Pr=0.71, Ra=100 and 1000, κ=1. The NC equations (1) is determined by exact velocities $u_{1}$ and $u_{2}$, pressure *p* and temperature *T*:

$$\begin{array}{c} u_{1}\left(x_{1},x_{2}\right)=\frac{y-0.1}{\sqrt{{\left(x-0.1\right)}^{2}+{\left(y-0.1\right)}^{2}}},\\ u_{2}\left(x_{1},x_{2}\right)=\frac{x-0.1}{\sqrt{{\left(x-0.1\right)}^{2}+{\left(y-0.1\right)}^{2}}},\\ p\left(x_{1},x_{2}\right)=\frac{1}{y+1.05}-\frac{\log(2.05)+\log(1.05)-2\log(0.05)}{3},\\ T\left(x_{1},x_{2}\right)=u_{1}\left(x_{1},x_{2}\right)+u_{2}\left(x_{1},x_{2}\right). \end{array}$$

We note that both velocity *u* and pressure *p* are continuous in the domain. However, it is clear that *u* and *p* are singular at the point (0.1,0.1) and along the line y=−1.05, respectively.

**Figure 2:**
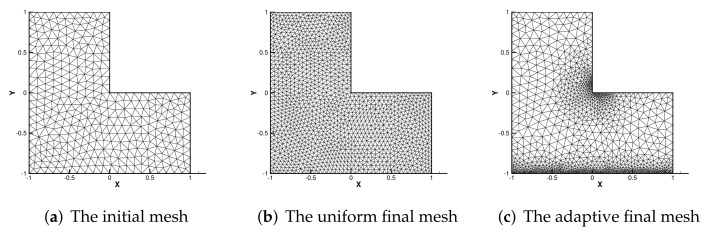
The initial mesh, uniform final mesh and adaptive final mesh.

To show the efficiency of the error estimator, we compare numerical results for adaptive refinements with those for uniform refinement. Figure 2 presents the initial mesh (left), the final uniform mesh (middle) and the final adaptive mesh (right). Table 4 and Table 5 show the numerical results for uniform/adaptive refinements with the recovery-based estimator $\eta_{r}$ .

According to in Figure 2c, we can see that near the singular point (0.1,0.1) of *u* and the line of y=−1.05 with the singularity of *p*, mesh refinement is carried out effectively, and the desired results are obtained, which shows that the adaptive mesh refinement is effective.

As can be seen from Table 4, Table 5 and Table 6, our adaptive method can obtain better accuracy with less vertex information, which is better than the uniform grid. For example, when Ra=100, to achieve accuracies of 0.1631 and 0.1665, the adaptive method only needs 1337 degrees of freedom, while the uniform grid requires 4282 degrees of freedom. When Ra=1000, the error is 0.2778 and 0.2602; the adaptive method only needs 3622 degrees of freedom, while the uniform grid needs 6172 degrees of freedom. This shows that our method is more efficient. Furthermore, as Ra becomes larger, our method calculates results that are superior to FEM.

### 5.3. Thermally Driven Flow

**Figure 3:**
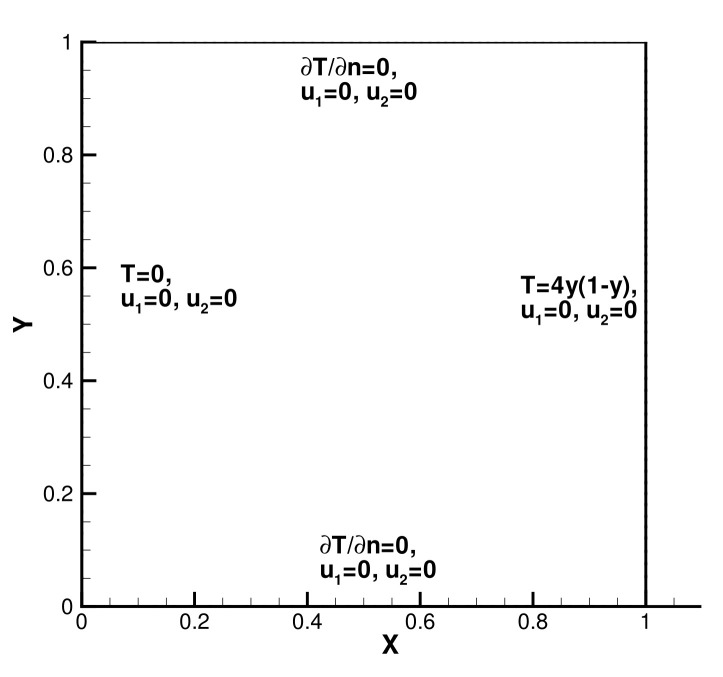
The model of thermally Driven Flow.

In the second numerical example, we consider the problem of square cavity flow without a true solution. As shown in Figure 3, the definition of the calculation area is $\Omega ={\left[0,1\right]}^{2}$, with Pr=0.71 and κ=1, and the boundary definition is as follows: the left boundary and the bottom are T=0, and the bottom edge is $\frac{\partial T}{\partial n}=0$. The rest is T=4y(1−y), and the speed is the 0 Dirichlet condition.

**Figure 4:**
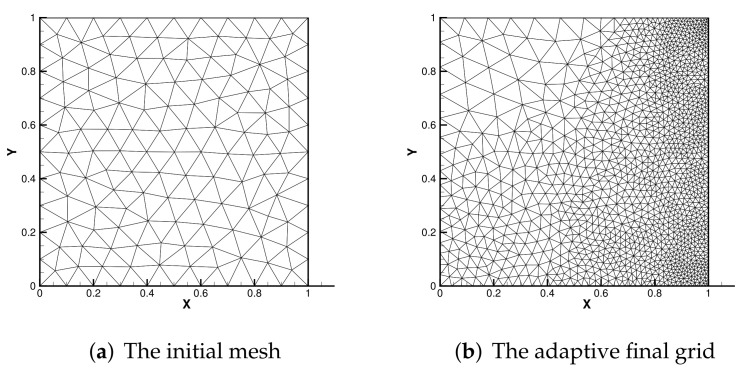
The initial mesh and adaptive final grid.

**Figure 5:**
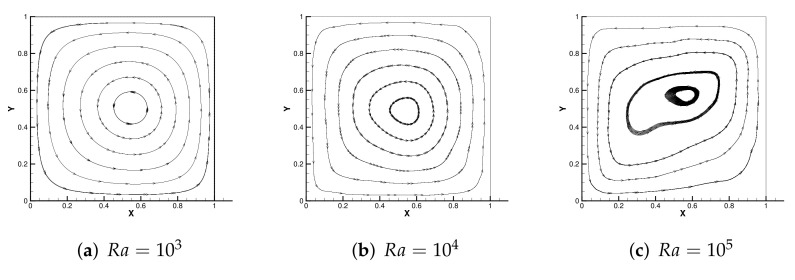
The streamline of the velocity with different Ra.

**Figure 6:**
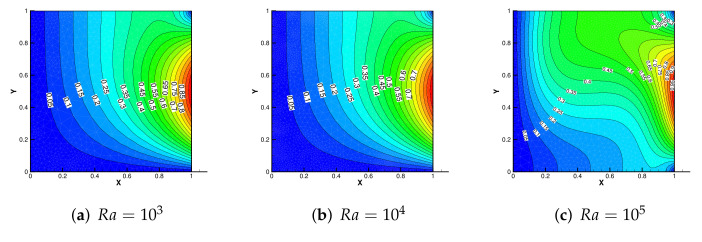
The isotherm diagram with different Ra.

**Figure 7:**
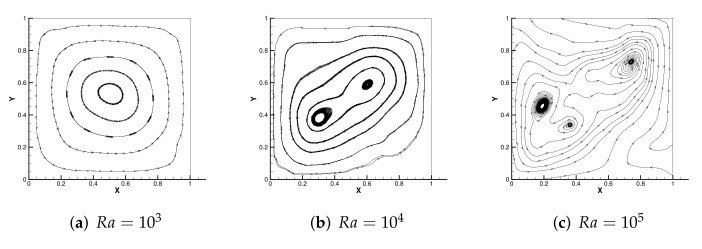
The streamline of the velocity without the defect-correction method with different Ra.

**Figure 8:**
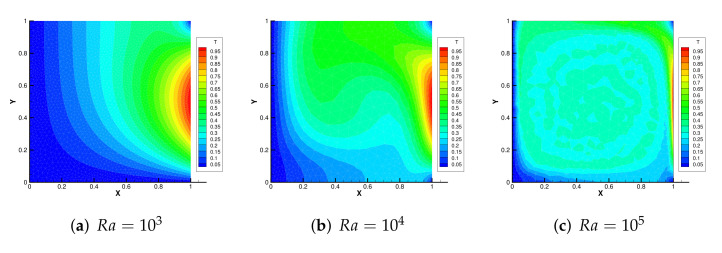
The isotherm diagram without the defect-correction method with different Ra.

**Figure 9:**
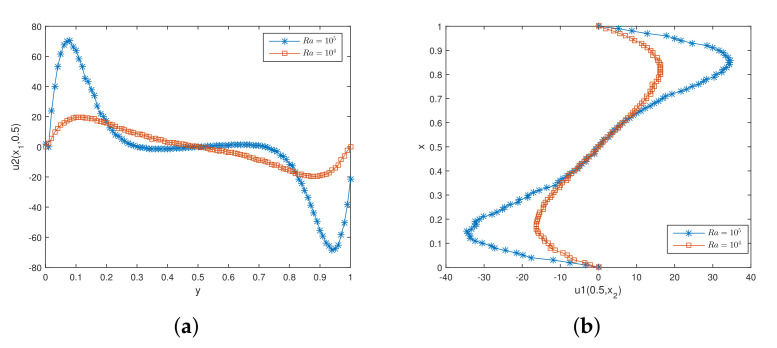
The values of vertical and horizontal velocity. (**a**) The vertical velocity at mid-height; (**b**) The horizontal velocity at mid-width.

Figure 4 shows the initial mesh and the adaptive encryption result grid. Figure 5 and Figure 6 are the velocity streamline and isothermal chart for different Rayleigh numbers: $10^{3},10^{4}$ and $10^{5}$. Figure 7 and Figure 8 present the results of the streamline of the velocity and the isotherm diagram calculated without the defect-correction method at different Ra numbers, and it can be seen that as Ra increases, the calculated results become more unstable [38]. In Figure 9, we also show the vertical velocity distribution at $x_{2}=0.5$ and the horizontal velocity distribution at $x_{1}=0.5$, which are very popular graphical illustrations in experimental studies of thermally driven cavities. We see that the profiles increasingly vary as the Rayleigh number increases, which is consistent with previous studies [5,7,39].

Table 7 and Table 8 present the peak values of vertical velocity at $x_{2}=0.5$ and horizontal velocity at $x_{1}=0.5$ for different Rayleigh numbers, respectively, where the number ’m’ in parentheses corresponds to the degree of freedom of the used mesh. It is easy to see that our results are concordant with benchmark data [5,7,39].

### 5.4. Bernard Convection Problem

**Figure 10:**
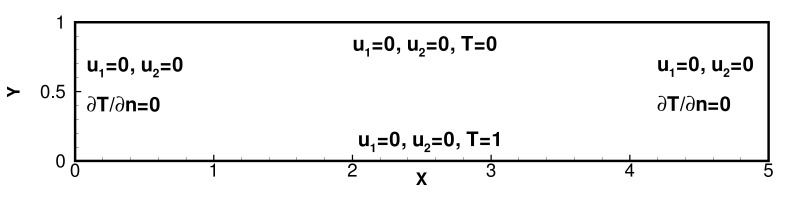
Physics model of Bernard convection problem.

In this experiment, we consider the domain Ω=[0,5]×[0,1] with the forcing f=0 and γ=0. Figure 10 displays the initial and boundary conditions for velocity *u* and temperature *T*: u=0 on ∂Ω, ∂T/∂n=0 on the lateral boundaries, with a fixed temperature difference between the top and bottom boundaries.

**Figure 11:**
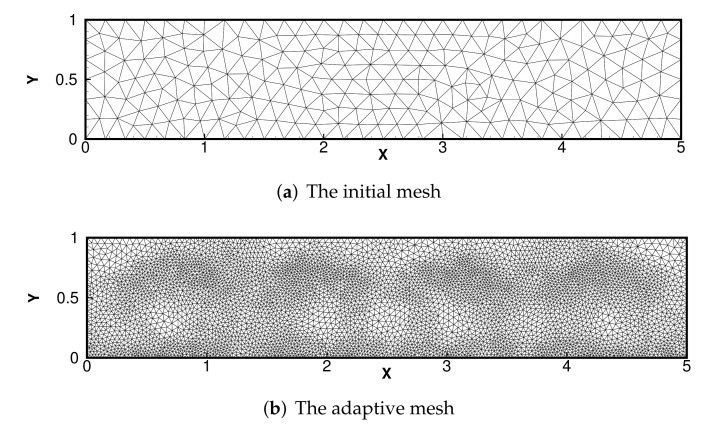
Initial mesh and final adaptive meshes.

**Figure 12:**
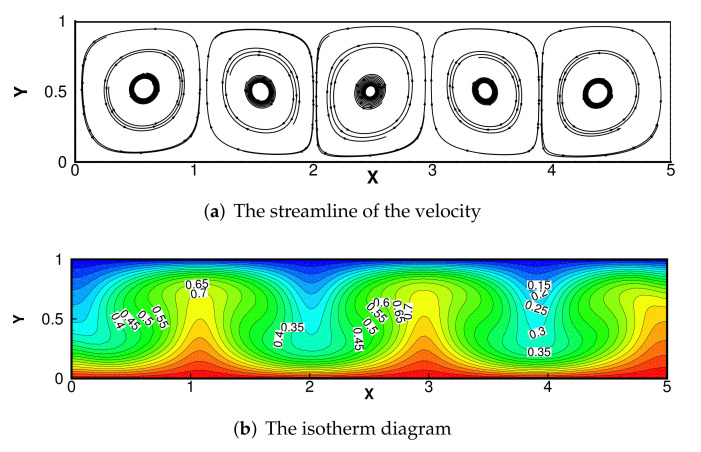
Calculation results of Bernard convection problem with $Ra=10^{4};Pr=0.71;\kappa =1$.

The results of numerical streamlines and numerical temperature are shown in Figure 11 and Figure 12 with Pr=0.71, κ=1 and $Ra=10^{4}$. The results coincide with the phenomenon described in the literature [38], indicating that our adaptive DCM is suitable.

## 6. Conclusions

In this work, a recovery-type error estimator for NC equations for the DCM finite element method is constructed based on the superconvergent patch recovery technique. Because the construction process is simple and does not involve the numerical solution of discrete NC equations, it is applicable to any stabilization method that can stabilize the lowest-order finite element to $P_{1}$–$P_{0}$–$P_{1}$. The results of numerical experiments fully verify the correctness of the theoretical analysis. The efficiency and stabilization of the new error estimator for the considered problems are proved. Furthermore, our method can be extended to more general fluid dynamics equations.

## Figures and Tables

**Table 1:** Adaptive results of smooth solution, where Pr=0.71;κ=1;Ra=1000.

*Level*	*DOF*	$e_{r}$	$e_{r}\mathrm{rate}$	$\eta_{r}$	$\eta_{r}\mathrm{rate}$	$I_{\mathrm{eff}}$
0	370	0.1317	-	0.1114	-	1.1824
1	691	0.0920	1.1482	0.0813	1.0056	1.1309
2	1395	0.0614	1.1523	0.0576	0.9820	1.0652
3	2773	0.0427	1.0528	0.0408	1.0018	1.0467
4	5494	0.0305	0.9848	0.0291	0.9898	1.0485

**Table 2:** Uniform mesh results of smooth solution, where Pr=0.71;κ=1;Ra=1000.

*Level*	*DOF*	$e_{r}$	$e_{r}\mathrm{rate}$	$\eta_{r}$	$\eta_{r}\mathrm{rate}$	$I_{\mathrm{eff}}$
0	450	0.1211	-	0.1133	-	0.9356
1	800	0.0928	0.9252	0.0886	0.8548	0.9547
2	1250	0.0758	0.9068	0.0732	0.8557	0.9644
3	5000	0.0415	0.8691	0.0409	0.8397	0.9855
4	6050	0.0383	0.8419	0.0379	0.7993	0.9819

**Table 3:** Comparison of DCM and non-DCM, with Pr=0.71; κ=1.

	*DOF*	Ra = $10^{3}$	*DOF*	Ra = $10^{4}$	DOF	Ra = $10^{5}$
DCM	2773	0.0408	2670	0.0653	2272	0.0923
NDCM	2392	0.1114	2677	13.91	NAN	NAN

**Table 4:** Uniform mesh and adaptive refinement results of DCM, where Pr=0.71;κ=1;Ra=100.

Level	Uniform Mesh			Adapt Mesh		
	***DOF***	$\eta_{r}$	$\eta_{r}\mathrm{rate}$	***DOF***	$\eta_{r}$	$\eta_{r}\mathrm{rate}$
0	676	0.3523	-	830	0.2547	-
1	1516	0.2627	0.7267	1337	0.1631	1.8698
2	2696	0.2043	0.8735	2344	0.1156	1.5798
3	4282	0.1665	0.8844	4558	0.0833	0.9855
4	6170	0.1419	0.8753	6315	0.0704	1.0135

**Table 5:** Uniform mesh and adaptive refine results of DCM, where Pr=0.71;κ=1;Ra=1000.

Level	Uniform Mesh			Adapt Mesh		
	***DOF***	$\eta_{r}$	$\eta_{r}\mathrm{rate}$	***DOF***	$\eta_{r}$	$\eta_{r}\mathrm{rate}$
0	676	0.7098	-	676	0.7098	-
1	1516	0.4955	0.8900	1423	0.4420	1.2728
2	2696	0.3805	0.9174	1952	0.3770	1.0065
3	4282	0.3059	0.9434	3622	0.2778	0.9879
4	6170	0.2602	0.9500	4970	0.2358	1.0362

**Table 6:** FEM with Pr=0.71;κ=1;Ra=100; Ra=1000.

***DOF***	Ra=100		Ra=1000	
	$\eta_{r}$	$\eta_{r}\mathrm{rate}$	$\eta_{r}$	$\eta_{r}\mathrm{rate}$
676	0.3610	-	0.4953	-
1516	0.2688	0.8900	0.3637	1.2728
2696	0.2086	0.9174	0.3061	1.0065
4282	0.1695	0.9434	0.2814	0.9879

**Table 7:** Comparison of maximum vertical velocity at $x_{2}=0.5$ with mesh size used in the computation.

*Ra*	Our Method	Ref. [5]	Ref. [39]	Ref. [7]
$10^{4}$	19.63(2445)	19.51(1681)	19.79(10,201)	19.74(1024)
$10^{5}$	70.22(2537)	68.22(6561)	70.63(10,201)	69.34(1024)

**Table 8:** Comparison of maximum horizontal velocity at $x_{1}=0.5$ with mesh size used in computation.

*Ra*	Our Method	Ref. [5]	Ref. [39]	Ref. [7]
$10^{4}$	16.37(2445)	16.18(1681)	16.10(10,201)	16.31(1024)
$10^{5}$	34.60(2537)	34.81(6561)	34(10,201)	35.83(1024)
